# The influence of journal submission guidelines on authors' reporting of statistics and use of open research practices

**DOI:** 10.1371/journal.pone.0175583

**Published:** 2017-04-17

**Authors:** David Giofrè, Geoff Cumming, Luca Fresc, Ingrid Boedker, Patrizio Tressoldi

**Affiliations:** 1 School of Natural Sciences and Psychology, Liverpool John Moores University, Liverpool, United Kingdom; 2 School of Psychology and Public Health, La Trobe University, Melbourne, Victoria, Australia; 3 Department of General Psychology, University of Padova, Padova, Italy; 4 Institute of Psychology, Health and Society, University of Liverpool, Liverpool, United Kingdom; Tilburg University, NETHERLANDS

## Abstract

From January 2014, *Psychological Science* introduced new submission guidelines that encouraged the use of effect sizes, estimation, and meta-analysis (the “new statistics”), required extra detail of methods, and offered badges for use of open science practices. We investigated the use of these practices in empirical articles published by *Psychological Science* and, for comparison, by the *Journal of Experimental Psychology*: *General*, during the period of January 2013 to December 2015. The use of null hypothesis significance testing (NHST) was extremely high at all times and in both journals. In *Psychological Science*, the use of confidence intervals increased markedly overall, from 28% of articles in 2013 to 70% in 2015, as did the availability of open data (3 to 39%) and open materials (7 to 31%). The other journal showed smaller or much smaller changes. Our findings suggest that journal-specific submission guidelines may encourage desirable changes in authors’ practices.

## Introduction

Recent years have seen a *crisis of confidence* in several scientific disciplines including psychological science and psychological research [1]. Data suggest that many, perhaps even most, psychological findings are not fully replicable [2], probably because of the endemic use of questionable research practices.

Questionable research practices can be broadly defined as a set of research practices that are typically employed with the purpose of presenting biased evidence in favor of an assertion [3]. There is extensive direct and indirect evidence that researchers commonly apply these practices in their work, which creates a number of biases [4,5]. These include, for example, the exclusion of data or relevant variables from the analyses, which is used to obtain desirable (typically significant) results [6,7], or the use of summation scores instead of the original measures [8]. Unfortunately, the use of these “*malpractices*” is very common [9] and different solutions have been proposed in response [9–15]. Namely, among other approaches, the *new statistics* have been adopted by a growing number of journals to deter authors from using questionable research practices.

Under the *new statistics*, authors have been discouraged from using the classical frequentist statistical approach of null-hypothesis significance testing (NHST), where an effect is only reported as “statistically significant” or not [16]. Instead, authors have been encouraged to report confidence intervals, to focus on the effect size [17]. Additionally, scholars have the option or are required to submit their research before beginning the study (pre-registration) [18] and to include the raw data (open data) or material (open material) [10,12,19]. Practices such as open data, open materials and pre-registration have proven to be extremely useful in discouraging the use of questionable research practices [20]. The *new statistics* approach also encourages the pre-registration and the publication of raw data and materials.

The response to this crisis of confidence has not been uniform and different guidelines have been introduced in response to these questionable research practices by different journals. These guidelines may also refer to a third party’s guidelines as a baseline, such as the American Psychological Association (APA)’s publication manual [21]. For example, the submission guidelines of *American Psychologist* only ask for effect sizes to be reported before directing authors to use APA style [22]. The Journal of Experimental Social Psychology also introduced new guidelines, which include sample size determination, data exclusion and variables exclusion (S1 File. JESP Editorial Guidelines). Similar guidelines were introduced for the Journals of the Psychonomic Society, which, for example, strongly discourage the use of NHST (S2 File. Journals of the Psychonomic Society Statistical Guidelines). Some other journals, including PLOS ONE, have made a requirement that data be openly available (S3 File. PLOS ONE data availability). Other journals, such as *Psychological Science* (*PS*), provide authors with complete journal-specific guidelines without deferring to an external body’s guidelines (S4 File. Psychological Science Statistical Guidelines). With the guidelines being similar in terms of content, it is worth investigating whether this key difference in how they are presented may influence authors’ adherence to the *new statistics*.

The Editor-in-Chief of *Psychological Science* (*PS*), an influential journal tied to the Association for Psychological Science (APS), introduced journal-specific submission guidelines which took effect from January 1^st^, 2014 [23]. Previously, the guidelines were not journal-specific and referred to the APA guidelines. The revised guidelines encourage the adoption of the so called *new statistics*, Research Disclosure, and Open Practice, and are as follows [24]:

1. Statistics section. Effective January 2014, Psychological Science recommends the use of the “new statistics”—effect sizes, confidence intervals, and meta-analysis—to avoid problems associated with null-hypothesis significance testing (NHST). Authors are encouraged to consult this Psychological Science tutorial by Geoff Cumming, which shows why estimation and meta-analysis are more informative than NHST and how they foster development of a cumulative, quantitative discipline.
2. Research Disclosure Statements section. For each study reported in your manuscript, check the boxes below to: (i) confirm that (a) the total number of excluded observations and (b) the reasons for making these exclusions have been reported in the Method section(s). [] If no observations were excluded, check here. []; (ii) confirm that all independent variables or conditions in all studies reported in the paper, whether they ended up being included in the analyses or not, have been reported in the Method section(s). [] If there were no independent variables or manipulations, as in the case of correlational research, check here. []; (iii) confirm that all dependent variables or measures that were analyzed for this article’s target research questions have been reported in the Methods section(s), (iv) specify how sample size in each study was determined and (b) your data-collection stopping rule have been reported in the Method section(s). []; (v) if sample size was based on past research, include the relevant reference information in your manuscript; and (vi) if sample size was based on power analysis, include in your manuscript the type of test (independent t-test, logistical regression, etc.) and the pertinent parameters: significance level (alpha), effect size (d), and power (1 –beta); all tests should be two tailed.
3. Open Practice section. Three open practices (or badges) were included in this section:
  (i) open data badge, which is earned for making publicly available the digitally-shareable data necessary to reproduce the reported result []; (ii) open materials badge, which is earned for making publicly available the digitally shareable materials/methods necessary to reproduce the reported results []; and (iii) preregistered badge, which is earned for having a preregistered design and analysis plan for the reported research and reporting results according to that plan. An analysis plan includes specification of the variables and the analyses that will be conducted.

Recent research indicates that the implementation of these new guidelines was found to be very effective in promoting the use of Open Practices from January 2012 to May 2015 [25]. However, it is still unclear whether or not this positive effect extends to other practices and whether the effects of these Open Practices were maintained after May 2015.

### Aims

The main aim of this preregistered study (https://osf.io/qhydk) was to analyze changes in the statistical reporting and the use of open practices in *Psychological Science* between January 1^st^ 2013 and December 31^st^ 2015, by comparing papers accepted under both the old, simply referring to APA rules, and the new guidelines. In *PS*, information about submission and acceptance of papers is always reported. We verified that all of the papers from 2013 and approximately half of those in 2014 had been accepted under the old guidelines, with the remaining papers from 2014 and the large majority of papers published in 2015 under the new guidelines.

To contextualize the extent to which practices changed across different psychology journals during this period, we also examined the *Journal of Experimental Psychology*: *General* (*JEP*: *General*). *JEP*: *General* was chosen for comparison as it is a prominent journal of the American Psychological Association (APA) that, similarly to *PS*, publishes empirical articles in a wide range of fields. *JEP*: *General* and *PS* are both considered to be top journals, with a high impact factor (i.e., 4.07 and 5.48 for the 2015). Both journals publish empirical research reports spanning the entire spectrum of the science of psychology. In addition, *JEP*: *General’s* guidelines refer to the statistical recommendations of the *APA Publication Manual*, which encourages the reporting of effect sizes, consideration of statistical power and use of confidence intervals, but makes no direct reference to open practices [21]. *JEP*: *General’s* guidelines also encourage the authors to withhold their data, but only for verification purposes (S5 File). Importantly, publishing guidelines for *JEP*: *General* link directly to the APA Publication Manual, rather than being separate written guidelines referencing the APA manual. It is also worth noting that in *JEP*: *General*, there were no changes in the chief editors or instructions to authors during the 2013–2015 period. Conversely, *PS* changed editorship when D. Stephen Lindsay become the Interim Editor-in-Chief in July 2015. However, this transition saw no substantive changes to the submission process, and we verified that almost all of the articles published in 2015 had been originally submitted while Eric Eich was still the Editor-in-Chief, with the new guidelines already well in place.

We hypothesized a positive change in the proportion of *PS* papers that used these guidelines after their implementation in the journal, in particular regarding Open Practices. Comparatively, we did not expect the same change to be observed with *JEP*: *General* papers.

## Materials and method

All papers published in *PS* and *JEP*: *General* between January 1^st^, 2013 to December 31^st^, 2015 were considered. It is worth mentioning that only the final published version, not an earlier version (if any) released online was considered. The sample included 305 *PS* papers from 2013, 246 from 2014, and 175 from 2015; and 91 *JEP*: *General* papers from 2013, 168 from 2014, and 92 from 2015. The database for the present study is available online (S6 File).

Inclusion and exclusion criteria. Only primary empirical papers reporting data from one or more empirical studies were included. Papers only reporting meta-analysis, narrative reviews, simulation, comments, theoretical studies were excluded. In particular, we excluded 6% of *PS* papers from 2013, 15% from 2014, and 19% from 2015; and 21% of *JEP*: *General* papers from 2013, 11% from 2014, and 13% from 2015. Please note that we excluded papers with a meta-analysis of multiple papers, while meta-analysis of multiple findings achieved in one paper were not excluded.

Scoring procedure and method (see also Table 1). Published journal articles, and the online supplemental material when available, were considered. A single occurrence of a practice anywhere in the published paper was sufficient for a coding of ‘Y’ (yes), indicating that this practice was adopted. Papers were examined for the following ten practices:

1. NHST. A *p* value was reported, whether exact (PE; e.g., *p* = .036) or relative (PA; e.g., *p* < .0*5*). We reported the overall proportion of papers adopting this practice and distinguished between papers that reported mainly the exact or relative value.
2. CI. A confidence interval was reported. CI counted all cases with any confidence interval. We reported the overall proportion of papers with at least one confidence interval for either standardized or unstandardized measures.
3. MA. Meta-analysis of multiple related results included in the paper was reported. We only included papers with more than one result related to the same empirical question.
4. CI_interp. A confidence interval was referred to in the discussion or interpretation of results, upon which data interpretation was explicitly based. For example, this would include a paper explicitly mentioning the width or the precision of the CI, a comparison between two or more CIs, or an overlapping between two intervals.
5. ES_interp. An effect size, either standardized or unstandardized, was referred to in the discussion or interpretation of results. We considered ‘effect size’ in the broad sense (17), including means, differences between means, percentages, and correlations, as well as Cohen’s *d*, *R*^2^, and *η*^2^. Papers were considered which included not only a dichotomous difference vs. no difference approach, but also those referring to the magnitude of the effect (e.g., small, large, strong etc.) or to the amount of explained variance. Effect size could be expressed in original units, or in some standardized or units-free form.
6. Sample_size. The authors described how sample size(s) were determined. For example, a power analysis - based on previous research, or on an estimated effect size–had been conducted. We used a very lenient approach, including all papers vaguely mentioning how the sample size was determined (e.g., the sample size was determined based on previous research, etc.).
7. Data_excl. The authors reported the criteria for data inclusion or exclusion—for example, the criteria for the exclusion of outliers.
8. Data. The paper carried the Open Data badge (see below), or stated where the data were available or how they could be obtained. We used a very lenient approach, including all the papers mentioning that data were available (e.g., data are available upon request).
9. Materials. The paper carried the Open Materials badge, or stated where details of the experimental materials and procedure could be obtained. We used a very lenient approach, including all the papers mentioning that materials were available (e.g., materials are available upon request).
10. Preregistered. The paper carried the Preregistered badge, or stated where a preregistered plan had been lodged in advance of data collection. Papers in this category typically included information about the number of the preregistration or where the preregistration is available.

**Table 1:** Checklist for study examination

Paper ID:			
**Statistics**	Value	Labels	Criteria for ‘Yes’ response
1-Null hypothesis significance testing	PE (p exact) PA (p relative)	NHST	At least one *p* value is reported; format: exact: e.g. *p* = 0.35; relative: e.g. *p* < .05.
2-Confidence intervals	Y	CI	At least one is reported; specify where: text, tables, figures;
**Statistical approach**			
3-Meta-analysis of reported data	Y/NA	MA	Authors meta-analyze results obtained in more than one reported experiment
4-Confidence intervals interpretation	Y	CI_Interpr	Authors explicitly refer to CIs in the comments and/or discussion of the results, e.g. *confidence intervals remained narrow enough…;*
5-Standaridzed or unstandardized effect size interpretation	Y	ES_Interpr	Authors explicitly refer to *ES*s in the comments and/or discussion of the results, e.g. *The effect size for the difference (11*.*94 percentage points) was large; effect sizes were moderate for comparisons with the low-intensity shock conditions*.
**Research practice disclosures**			
6-Sample size determination	Y	Sample_size	Authors explicitly clarify how they determined the sample size(s), e.g. power estimate, previous studies, etc.
7-Sample size stopping rule	Y	Data_excl	Authors explicitly declare if and which stopping rule where adopted or the criteria to exclude data and/or manage outliers
**Open Practices**			
8-Data availability	Y	Data	Authors explicitly give information on how the data may be obtained, e.g. posted in a repository; author email, etc.
9-Materials availability	Y	Mater	Authors explicitly give information on how to obtain the materials, equipment and/or software used in the study
10-Preregistered design & analysis plan	Y	Prereg	Authors explicitly declare where the study was preregistered

The three badges (i.e., Data, Materials and Preregistration) are described in detail by the Center for Open Science (tiny.cc/badges; accessed by June, 2016). For *PS*, these badges were certified by an “earned badge” from the Open Science Framework (https://osf.io/tvyxz/wiki/home; accessed June 2016). It is noteworthy that the while the badges were created by the Center for Open Science and the criteria maintained on the Open Science Framework, the editorial team at *PS* is responsible for awarding manuscripts with any of the three open science badges. For *JEP*: *General*, badges were not available, but we considered whether or not authors clearly indicated how to obtain the data and/or the materials.

## Data analysis

### Scoring method and reliability

Papers were examined for the presence of each of the ten practices. For each of them, the score could be “Y” (yes) if present.

Papers were divided by the authors and independently scored. The authors are experienced researchers with good knowledge of the statistics examined. Secondly, a random sample comprising ten percent of the papers was scored independently by both raters to test inter-rater reliability. Mean inter-rater reliability was 90% across all ten variables, ranging from 99% for type of *p* value to 85% for CI and ES interpretation. Discrepancies were solved by discussion.

Only descriptive statistics are reported given that they refer to the whole populations of studies. For each of the ten practices analyzed, the number of papers including a practice was expressed as a proportion of the total number of papers. Only for the meta-analysis (MA) did we exclude from the total papers those for which the meta-analysis criterion was not applicable (NA), namely papers with a single study. The checklist for study examination is presented in Table 1.

### Changes with respect to the preregistered study

We considered all of the papers that appeared in *Clinical Psychological Science* (*CPS*) between 2013 and 2015. However, the comparison with *CPS* was problematic. In fact, *CPS* is a very young journal and publishes articles having to do with clinical psychology, unlike *Psychological Science* and the *Journal of Experimental Psychology*: *General*. Therefore, we decided to report the *CPS* (S7 File) but to avoid to comparing this journal with *Psychological Science* or the *Journal of Experimental Psychology*: *General* (see S1 Text. Changes with respect to the preregistered study).

## Results and discussion

### Changes in practices over time

**Fig 1:**
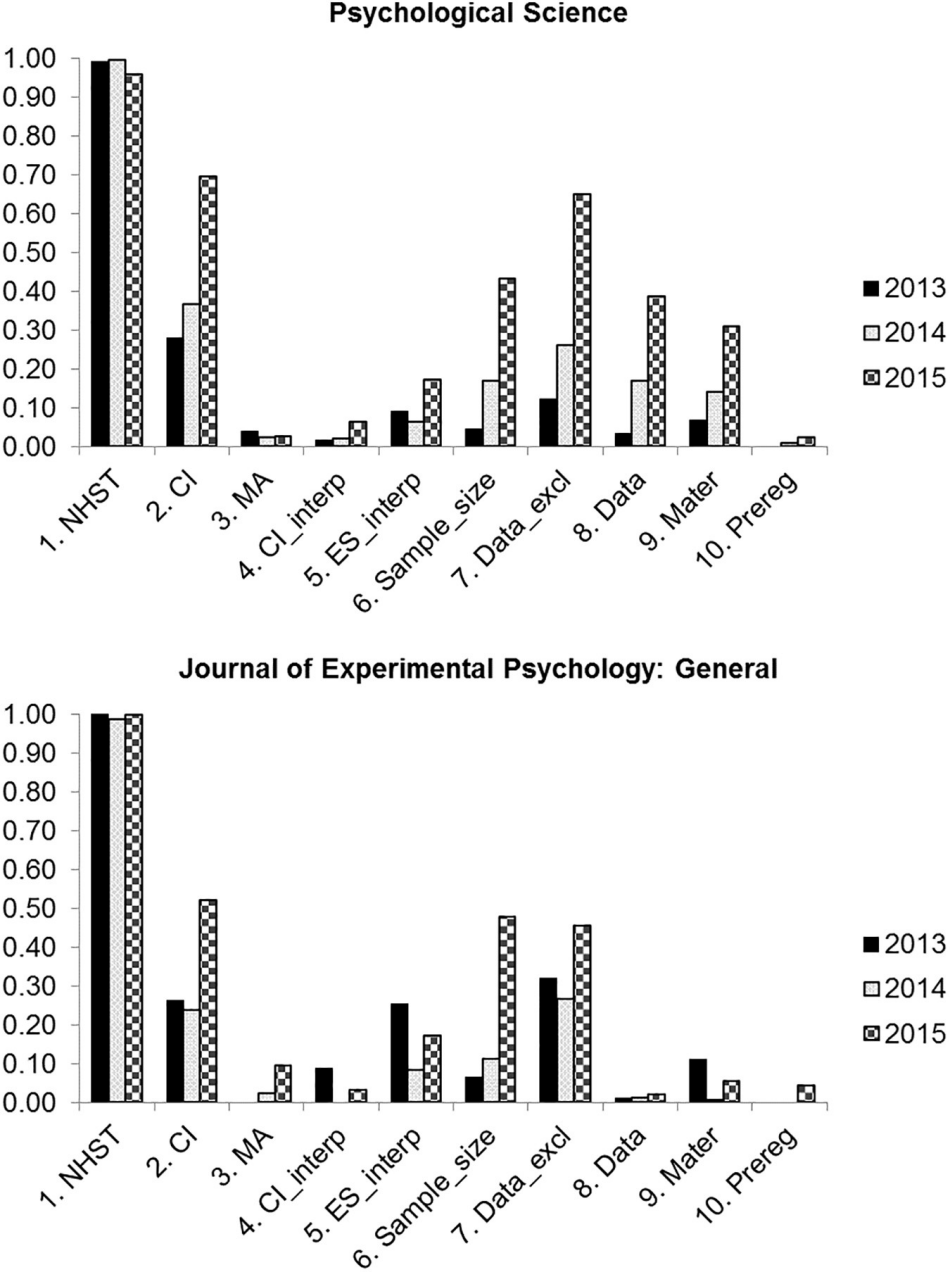
Proportions of papers in Psychological Science, and the Journal of Experimental Psychology: General, from the 2013 to the 2015, for each of the ten practices. NHST = null hypothesis significance testing; CI = confidence intervals; MA = meta-analysis; CI_interp = confidence intervals interpretation; ES_interp = effect size interpretation; Data_excl = exclusion criteria reported; Material = additional materials availability; Prereg = preregistered study. For the meta-analysis (MA) the proportion of papers with more than one related study, i.e. have potential for MA, was considered.

**Fig 2:**
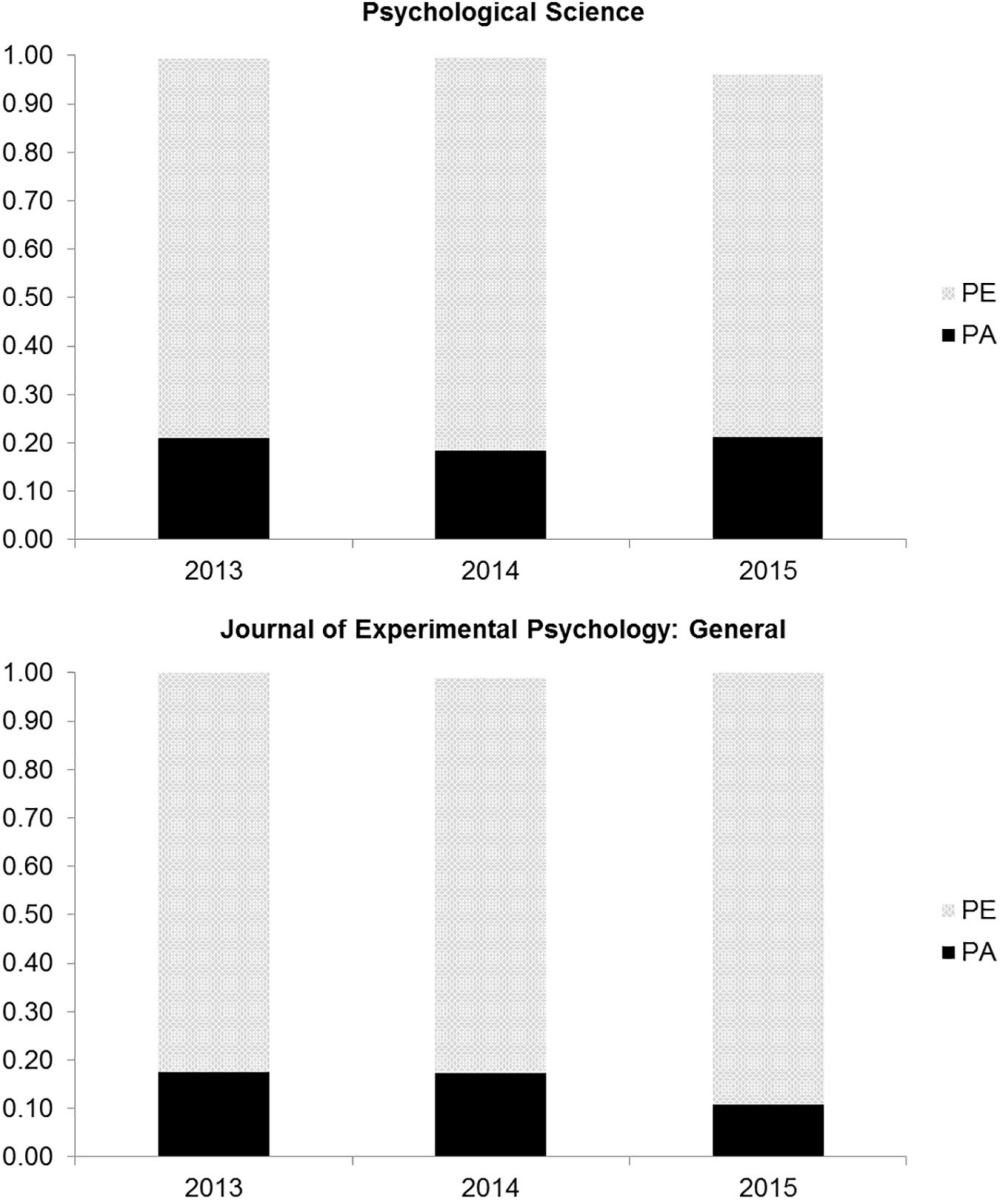
Proportions of papers with a PA (p relative) or a PE (p exact), in Psychological Science, and the Journal of Experimental Psychology: General from the 2013 to the 2015.

Fig 1 shows the proportion of various practices in each of the three years for *PS* and *JEP*: *General*. The incidence of exact and relative *p* values are reported in Fig 2. Please note that we did not perform any statistical test of the difference over time and between journals because we considered the entire population (Table 2).

**Table 2:** Proportions of papers in *Psychological Science* and the *Journal of Experimental Psychology*: *General* from the 2013 to the 2015, for each of the ten practices.

Journal	PS			JEP: General		
Year	2013	2014	2015	2013	2014	2015
1. NHST	0.99	1.00	0.96	1.00	0.99	1.00
2. CI	0.28	0.37	0.70	0.26	0.24	0.52
3. MA	0.04	0.02	0.03	0.00	0.02	0.10
4. CI_interp	0.02	0.02	0.06	0.09	0.00	0.03
5. ES_interp	0.09	0.07	0.17	0.25	0.08	0.17
6. Sample_size	0.05	0.17	0.43	0.07	0.12	0.48
7. Data_excl	0.12	0.26	0.65	0.32	0.27	0.46
8. Data	0.03	0.17	0.39	0.01	0.01	0.02
9. Mater	0.07	0.14	0.31	0.11	0.01	0.05
10. Prereg	0.00	0.01	0.02	0.00	0.00	0.04

NHST = null hypothesis significance testing; CI = confidence intervals; MA = meta-analysis; CI_interp = confidence intervals interpretation; ES_interp = effect size interpretation; Data_excl = exclusion criteria reported; Material = additional materials availability; Prereg = preregistered study. For the meta-analysis (MA) the proportion of papers with more than one related study, i.e. having potential for MA, was considered.

In both journals over all three years, virtually all empirical papers used NHST, with about 80% reporting the *p* exact values (Fig 2). This is particularly interesting, because it confirms that although the APA (21) advises to report the exact *p* value for values greater than .001, many authors are still failing to do so, including very imprecise estimates (e.g., *p* < .05, or all test < .05 are not included). This shows that although the use of exact *p* values has recently increased, e.g., in the 1985–2013 period, the use of relative (or inexact) *p* values is still present [26]. Despite the fact that the statistical guidelines of both journals did not encourage the use of NHST, dichotomous decision making based on NHST remained very high.

### Changes in Psychological Science

Overall, in *PS* there was a positive change in all practices except NHST and MA. In particular, from 2013 to 2015, the inclusion of CIs increased from 28% to 70% (i.e., about a 41% increase). Among the other relevant positive trends, there was an increase from 5% to 43% (i.e., about a 38% increase) of mention of how the sample(s) size was determined (a striking increase with respect to the 5%, observed in 2013) [27] and an increase, from 12% to 65% (i.e., about a 53% increase), in the number of studies providing information on the criteria adopted for data inclusion/exclusion.

As for the use of the three open access practices, there was an increase from 3% to 39% (i.e., about a 36% increase) of the availability of data and an increase from 7% to 31% (i.e., about a 24% increase) of other materials. The use of a meta-analysis (MA) did not see an evident increase, with less than 5% of the papers that reported more than one related study reporting one. The increase in preregistration was also very small (only about 2% for 2015).

### Comparison with JEP: General

In general, it seems that CI use and sample size justification are increasingly being adopted in *JEP*: *General* [21]. There has also been a positive increase in the use of CIs, from 26% to 52%, a positive trend in the justification of sample size, from 7% to 48%, and in the disclosure of the criteria used to exclude the data, from 32% to 46%. A modest but positive increase in the application of open practices is observed in this journal. Comparing *PS* and *JEP*: *General*, the statistical practices, with some exceptions, improved from 2013 to 2015. It is also worth mentioning that half of the papers published in *JEP*: *General* reported two or more studies, making it possible to perform a meta-analysis. In fact, the number of meta-analyses increased from virtually zero to 10% from 2013 to 2015.

Comparing *PS* with *JEP*: *General*, statistical practices improved overall in both journals, although the changes in *PS* were typically greater, particularly in the open practices.

### Have there been improvements in the statistical reporting?

For *PS* and *JEP*: *General*, the answer is a clear yes. It can be suggested that *PS*’s and APA’s statistical guidelines are, at least in part, involved in the increased use of CIs, in the justification of sample sizes and the disclosure of the data exclusion criteria. However, it is worth mentioning that the APA guidelines came out in 2010, and changes in *JEP*: *General* could also be due to general changes in the discipline, or even secondary influence from the new *PS* guidelines.

Moreover, there were larger and broader changes in *PS* practices. One possible implication of this is that journal-specific submission guidelines can influence authors’ and reviewers’ statistical practices. However, the present study is correlational and caution should be exercised in drawing conclusions.

### Does the improvement in the use of some statistical practices represent a substantial change?

It is uncertain whether the improvement in the use of some statistical practices represents a substantial change. On one hand, CI intervals, sample size determination and data exclusion practices have improved considerably. However, we believe that there is still an overreliance on interpreting results based solely on NHST and on *p*-values. In addition, the explicit interpretation of CIs and ESs, even if it seems somewhat improved in *PS*, is modest and is similar between *PS* and *JEP*: *General*. This is consistent with previous evidence indicating that there was a strong increase in the use of NHST in recent years (up until 2013) in both journals [26]. In fact, in the period between 2013 and 2015, we found that almost the totality of papers in this journal report at least one *p*-value. This finding may indicate that the *new guidelines* only superficially change the practices, while many authors still rely heavily on a dichotomous statistical approach. Finch et al. [28] observed this same effect by examining 696 *Memory and Cognition* articles published before, during, and after Geoffrey Loftus’ editorship, during which the avoidance of NHST was strongly encouraged. They concluded that even strong editorial encouragement is not sufficient to change long-entrenched practices. To see more substantial improvements may require a change in how statistics and research methods are taught, as well as increased availability of guidelines, in order to make the adoption of practices easy [29]. In fact, the use of Open Practices in *PS* in was similar between 2015 and 2016 and did not increase substantially: 38% Open Data (about -1% compared to 2015), Open Materials 27% (about -4% compared to 2015), and 3% Preregistration 3% (about +0.4% compared to 2015). This latter finding can be used as a confirmation that only changing the guidelines may be insufficient.

However, by shifting many incentives underlying long-entrenched scientific practices to make the adoption of new practices easy, there is a possibility that substantial change can occur; and in fact, initiatives to promote this shift have been occurring in the community (e.g., preregistration / Preregistration Challenge, TOP Guidelines, Peer Reviewers’ Openness initiative).

### Have there been improvements in open practices?

Improvements in open practices in *PS* seem to be limited to data availability (39% in 2015) and to the materials (31% in 2015). Preregistration seems unaffected by the new submission guidelines, and the 2% observed in 2015 is not a very good result. It is worth noting, however, that the preregistration must occur before data collection and before submission to the journal. Therefore, reporting may come a year or more later, i.e., outside of the time-period considered in the present report. In addition, many authors may be still unaware of the preregistration opportunity. It can be argued that is appreciably harder to preregister a study than it is to share data and materials. However, the launch of the Preregistration Challenge (https://cos.io/prereg), which aims to reward 1000 researchers with $1,000 for publishing research whose study and analysis plans were preregistered on the Open Science Framework, could be a strong incentive for the use of this practice. It is hoped that this percentage will increase steadily in the future. In fact, the number of preregistrations preserved on the Open Science Framework (https://osf.io, accessed June 2016) is quite large (4628) and very promising-–although we did not control for double counting, or counting of mere registration of research protocols.

In *JEP*: *General*, the percentages of studies providing, or mentioning the availability, of data or materials never exceeded 11%, with no signs of positive trends. This finding does not necessarily mean that the authors did not make their data or materials available upon direct request, but it is consistent with journal submission guidelines that do not specifically encourage these practices. Similarly, very few studies were preregistered. However, an increasing number of journals are now offering the option to submit pre-study peer review (i.e., ‘registered review’ or ‘preregistered review’), which can be considered a step beyond merely reporting that a study had been preregistered (see an updated list at http://tiny.cc/sc8t6x) (e.g., S2 Text).

### Lesson learned

Our findings provide convergent support to the initiatives that emphasize the critical role of journal editors and reviewers in the promotion of reforms in scientific practices. Journal editors and reviewers are crucial in verifying that the practices proposed are adopted by the authors. Among the ongoing initiatives, the Transparency and Openness Promotion (TOP) guidelines (available here https://cos.io/our-services/top-guidelines/) [30], are directed to the editors of scientific journals and encourage adoption of guidelines with the aim of increasing the transparency, openness, and reproducibility of published studies.

Another recent initiative is the Peer Reviewers' Openness Initiative (https://opennessinitiative.org; accessed June, 2016) [31]. Researchers supporting the initiative declare that they will only review manuscripts in which data and other materials are open-access.

### Limitations

Choice of comparison journal. It is difficult to make a direct comparison between the submission guidelines of two journals as there are numerous factors to consider. Journals may be influenced by the publisher or society to which they are associated, the subject matter, the technical aspects of submission, or age and pedigree of the journal.

*JEP*: *General* provided a comparison as a journal that did not have detailed statistical guidance. However, for balance it also would have been useful to include an established journal with statistical guidance. For example, *Psychonomic Bulletin & Review* has extensive statistical guidance in their Instructions for Authors and it would be interesting for future studies to also include this journal [32].

Time-frame. Our analysis was limited only to three years, from 2013 to 2015. In fact, *JEP*: *General* has a long history, more than a century, while *PS* is about 26 years old. In this paper we were only interested in recent changes. However, it would also be interesting to evaluate the statistical practices of these journals using a wider time-frame, e.g., 1985–2013 used in other studies [26]. Additionally, it should be noted that pre-registered studies may be ongoing for a longer period of time before they reach the publication stage. Therefore, an increased incidence of pre-registered studies may not be immediately visible in the time frame that we chose.

Confidence intervals and effect size interpretation. We believe that it is difficult to establish whether an author has interpreted an ES or a CI. We only coded CI_interp or ES_interp if the authors explicitly interpreted the CI or the ES. However, we cannot exclude the possibility that the proportion of papers falling within these two categories would have increased using a more lenient approach.

Sample size. We used a very lenient approach for sample size determination. It can be argued that research can be rather vague on the sample size determination (e.g., sample size was determined based on previous research etc.), and many authors overlook the importance of performing a power analysis before collecting data [33]. To address this point, we decided to consider only papers in which an *a priori* power analysis was clearly specified, based on previous data or on effect sizes. As a result, the percentage of papers included in this category diminished considerably: only 1% of *PS* papers in 2013, 4% in 2014, and 20% in 2015 conducted an *a priori* power analysis; compared to 2% of *JEP*: *General* papers in 2013, 8% in 2014 and 33% in 2015. These results confirm that use of prospective statistical power is increasing in both journals, although the number of papers using this approach is still quite low with only a fifth or a third of papers in *PS* and *JEP*: *General*, respectively.

Data. Our results on open data sharing rates in *PS* are consistent with a recent paper by Kidwell and coauthors [25]. In their paper, which was limited to January 2012—May 2015 and to open practices only, they found that when authors earn a badge for posting data it is usually less than fully complete, fine-grained, and raw, and only 1% present complete data. In fact, the degree of detail varies widely, but, typically, item-level details are lost and only summary values (e.g., mean percent correct in each condition) by subject are given. Notably, we were very lenient in our inclusion criteria, including also papers with generic statements such as “data are available upon request.” However, such promises are often hollow as the authors are often unwilling to share their data [34,35]. In fact, when we used a more rigorous approach, scoring as a “yes” only when the raw data were actually shared via the publisher website or via a robust repository, the situation was different. As for *PS*, data were available for 0.9% of papers in 2013, 13% of papers in 2014, and 34.3% of papers in 2015. For *JEP*: *General*, data were available for 0 of papers in 2013, 0.6% in 2014, and 2% in 2015. It is noteworthy that, unfortunately, on several occasions it was not possible to access the data for papers awarded with a badge in *PS*. This is particularly unfortunate and confirms the importance of depositing data on robust and reliable repositories (e.g., on the Open Science Framework). Finally, though there was a rapid and tremendous increase in the number of papers sharing their data between 2013 and 2015, more recently (i.e., in 2016) this trend stopped, confirming that badges alone are not sufficient.

Materials. We used a very lenient approach for the materials availability. However, we recognize that the majority of researchers fail to share data after publication [36], and often materials are unavailable after request [37]. When we used a more rigorous approach, scoring as a “yes” only when the raw materials were actually shared via the publisher website or via a robust repository (March, 2017) the percentage of papers included in this category was typically reduced. For *PS*, materials were available for 1.6% of papers in 2013, 13% of papers in 2014, and 28% of papers in 2015. For *JEP*: *General*, materials were available for 2% of papers in 2013, 0 in 2014, and 4% in 2015.

### Summing up

To sum up, we cannot assess the extent that observed changes were caused by guideline changes, but it seems that changes in guidelines may be useful although not sufficient. Changing guidelines may be effective for some practices but rather less so for others. Substantial innovation in science practice seems likely to require multiple strategies for change, including, in particular, the coordinated efforts of journal editors, reviewers and authors.

Broadly speaking, it could be suggested that many authors take a “bare-minimum” approach, therefore journal-specific submission guidelines may have a greater impact than reference to an external source, such as APA. Consequently, it may be in a journal’s best interest and best practice to give authors specific directions on reporting of statistics and use of open practices even when these are nearly identical to, and readily available from, other sources.

## Supporting information

S1 File JESP Editorial Guidelines. (PDF)

S2 File New Statistical Guidelines for Journals of the Psychonomic Society. (PDF)

S3 File PLOS ONE data availability. (PDF)

S4 File Psychological Science Statistical Guidelines. (PDF)

S5 File Journal of Experimental Psychology: General Guidelines. (PDF)

S6 File Complete Database. (CSV)

S7 File Clinical Psychological Science results. (PDF)

S1 Text Changes with respect to the preregistered study. (PDF)

S2 Text Examples of journals offering the option to submit pre-study peer review. (PDF)
