# Supplementary material for: The influence of journal submission guidelines on authors' reporting of statistics and use of open research practices
Source: PLoS One. 2017 Apr 17;12(4):e0175583. doi: 10.1371/journal.pone.0175583 (PMC5393581; doi:10.1371/journal.pone.0175583)
Supplement: S1 File — (PDF) [file pone.0175583.s001.pdf]

S1 File. JESP Editorial Guidelines

Retrieved from: https://www.journals.elsevier.com/journal-of-experimental-social-psychology/news/jesp-editorial-guidelines/ (Accessed March 2017)

Version of 15 January 2016.

These policies build on and replace the requirements and guidelines of June 2014. They have been agreed upon after discussion with the JESP associate editorial staff for 2016-18. Points 1 and 2 are requirements for manuscripts; points 3 and 4 are new policies; and points 5 through 9 explain the editorial staff's points of view on evidence and some specific statistical methods.

1. All manuscripts must include a report of the following items:

Explain how sample size was determined, including whether and how looking at the results influenced the collection of additional data. Reporting statistical power (together with the basis for expected effect size) is welcome, but not required.

If participants were excluded from the study, report how many, how they were distributed across conditions, and the reasons for exclusion.

Disclose the existence of all variables and conditions that were part of the study. These can be summarized, or put in a footnote or supplementary material in the case of large numbers of variables, but there should be enough information to judge whether they are potentially theoretically relevant or not.

Affirm the above two disclosures positively (that is, with a statement such as "We report all measures, manipulations, and exclusions in these studies.")

Report procedures in sufficient detail to allow close replication by an independent lab. This may include citations of method details found in other published, peer-reviewed (or open access) sources. Materials are not required to be provided verbatim, but should be summarized with examples. To stay within the word limit of a report, necessary details can be included in Supplementary Materials.

2. All manuscripts should report complete statistics relevant to the analyses at hand, using supplementary materials if needed:

cell means, SD and n for experimental designs

correlations between variables for multivariate designs including regression and repeated-measures

inferential statistics with exact p-values and effect sizes regardless of significance level.

If figures use error bars, these should be explained in a caption (e.g., standard error, 95% confidence interval, etc.)

If meeting any of these requirements proves impractical, the authors should explain why.

3. FlashReports will now follow similar standards of evidence and potential topics as the other forms of articles, but will still be distinguished by a brief 2500 word limit and streamlined review process. The short format encourages research reports with background ideas and methods that do not require a great deal of explanation, but it does not mean that papers should be “short” on theoretical relevance or amount of evidence. Method and analysis details not crucial to the narrative of the paper should still be reported in Supplementary Materials.

4. Soliciting reviews of revised manuscripts is now the exception, not the rule. Associate Editors will typically come to a decision on a revised manuscript based on its capacity to address the reviewers’ comments, unless expert opinions on technical matters are required. We expect this will make the review process faster and more efficient.

5.Our standards for articles rest on a tripod of strong theory, strong methods, and strong statistical evidence for the points being made. Deficiencies in one of these areas can, to some extent, be compensated by strengths in the other two.

a. In particular, our view of strong statistical evidence departs from the previous unwritten standard of “multiple studies, each with a significant effect.” Instead, strong statistical evidence for a central positive claim now rests more upon showing a plausible pattern of results, usually from multiple comparable studies, whose total joint probability under the null would be about $p = .01$ or less (e.g., using Fisher’s method to aggregate p values from results in the same direction, or meta-analysis otherwise). However, we emphasize that this view is not a strict criterion. It does not prohibit publication of less strong results if theory and methods are both strong, nor is it a guarantee of publication if the article has other limitations. For example, although we continue to discourage submission of single-study articles because they often do not provide enough development and evidence for an idea, we are willing to consider them if based on good theory and exceptionally strong methods (possibly including high power to detect a small-to-medium effect size, pre-registered methods and analyses, unusual or highly representative samples).

b. Within an article, individual studies with good theoretical and methodological connections to the others need not be statistically significant if they contribute to a strong overall pattern, a precise estimate, and/or a complete and open reporting of a program of research. Article-wise meta-analytic estimates are encouraged to aggregate the findings of multiple studies.

c. Bayesian analyses with well-justified and well-explained priors can be used to assess evidence for reasonable null and alternative hypotheses.

d. A pattern of multiple central results that are all below, but close to, $p = .05$, although they might have arisen by chance, also can be said to show low statistical robustness. Ideally, confidence in such a set of results can be bolstered by pre-registered studies or other methodological assurances against selective reporting.

e. As before, we welcome rigorously conducted replication articles that meet the criteria described in Brandt, IJzerman, et al. (2014).

6. Authors are encouraged to present exploratory work openly. It is deceptive to present hypotheses as perfectly precognitive when they aren't (i.e., avoid HARKing; Kerr, 1998). It is OK for authors to admit they entertained multiple hypotheses, based on multiple theoretical perspectives, without coming down on the side of any one of them – or even to admit that they came down on the side of the wrong one! Put another way: an initial study, with an uncertain or novel prediction, should be treated as exploratory regardless of what story the authors choose to tell. Confidence in the results depends on sound methods, sample size, and consistent replication. Honest distinctions between exploratory and confirmatory work can be reported concisely without turning it into a long-winded "intellectual odyssey."

7. Although mediation analyses are used in many, if not most, recent JESP articles, we urge greater caution in using and interpreting this technique (cf. Fiedler, Schott & Meiser, 2011; Kline, 2015; Spencer, Zanna, & Fong, 2005). As before, we see little value in mediation models in which the mediator is conceptually very similar to either the predictor or outcome. Additionally, good mediation models should have a methodological basis for the causal assumptions in each step; for example, when the predictor is a manipulation, the mediator a self-reported mental state, and the outcome a subsequent decision or observed behavior. Designs that do not meet these assumptions can still give valuable information about potential processes through correlation, partial correlation, and regression, but should not use causal language and should interpret indirect paths with caution. We reiterate that mediation is not the only way to address issues of process in an experimental design.

8. Arbitrary use of covariates can be used to engineer significant results. Therefore, covariates need to be justified as a response to a clear threat to validity. Reporting the uncovaried analysis can help clear this up (e.g., in a Footnote).

9. Interaction effects on their own are not sufficient; they must be decomposed with simple effects analysis (Aiken & West, 1991) or other means. At the same time, the direction and significance of simple effects are influenced both by interactions and main effects; therefore, it is not always necessary to “validate” an interaction by showing that both simple effects are significant (Petty, Fabrigar, Wegener, & Priester, 1996).

References

Aiken, L. S., & West, S. G. (1991). Multiple regression: Testing and interpreting interactions. Newbury Park, CA: SAGE Publications.

Brandt, M. J., IJzerman, H., Dijksterhuis, A., Farach, F. J., Geller, J., Giner-Sorolla, R., Grange, J. A., Perugini, M. Spies, J. R., & Van’t Veer, A. (2014). The replication recipe: What makes for a convincing replication? Journal of Experimental Social Psychology, 50, 217–224.

Fiedler, K., Schott, M., & Meiser, T. (2011). What mediation analysis can (not) do. Journal of Experimental Social Psychology, 47(6), 1231–1236.

Kerr, N. L. (1998). HARKing: Hypothesizing after the results are known. Personality and Social Psychology Review, 2(3), 196–217.

Kline, R. B. (2015). The mediation myth. Basic and Applied Social Psychology, 37(4), 202–213.

Petty, R. E., Fabrigar, L. R., Wegener, D. T., & Priester, J. R. (1996). Understanding data when interactions are present or hypothesized. Psychological Science, 7(4), 247–252.

Spencer, S. J., Zanna, M. P., & Fong, G. T. (2005). Establishing a causal chain: Why experiments are often more effective than mediational analyses in examining psychological processes. Journal of Personality and Social Psychology, 89, 845–851.
