# Supplementary material for: The influence of journal submission guidelines on authors' reporting of statistics and use of open research practices
Source: PLoS One. 2017 Apr 17;12(4):e0175583. doi: 10.1371/journal.pone.0175583 (PMC5393581; doi:10.1371/journal.pone.0175583)
Supplement: S2 File — (PDF) [file pone.0175583.s002.pdf]

S2 File. New Statistical Guidelines for Journals of the Psychonomic Society

Retrieved from: http://www.springer.com/psychology?SGWID=0-10126-6-1390050-0 (Accessed March 2017)

The Psychonomic Society's Publications Committee and Ethics Committee and the Editors in Chief of the Society's six journals worked together (with input from others) to create these guidelines on statistical issues. These guidelines focus on the analysis and reporting of quantitative data. Many of the issues described below pertain to vulnerabilities in null hypothesis significance testing (NHST), in which the central question is whether or not experimental measures differ from what would be expected due to chance. Below we emphasize some steps that researchers using NHST can take to avoid exacerbating those vulnerabilities. Many of the guidelines are long-standing norms about how to conduct experimental research in psychology. Nevertheless, researchers may benefit from being reminded of some of the ways that poor experimental procedure and analysis can compromise research conclusions. Authors are asked to consider the following issues for each manuscript submitted for publication in a Psychonomic Society journal. Some of these issues are specific to NHST, but many of them apply to other approaches as well. We welcome feedback regarding these guidelines via email to info@psychonomic.org with the Subject heading "Statistical Guidelines."

1. It is important to address the issue of statistical power. Statistical power refers to the probability that a test will reject a false null hypothesis. Studies with low statistical power produce inherently ambiguous results because they often fail to replicate. Thus it is highly desirable to have ample statistical power and to report an estimate of a priori power (not post hoc power) for tests of your main hypotheses. Best practice when feasible is to draw on the literature and/or theory to make a plausible estimate of effect size and then to test a sufficient number of participants to attain adequate power to detect an effect of that size. There is no hard-and-fast rule specifying "adequate" power, and Editors may judge that other considerations (e.g., novelty, difficulty) partially offset low power. If a priori power cannot be calculated because there is no estimate of effect size, then perhaps the analysis should focus on estimation of the effect size rather than on a hypothesis test. In any case, the Method section should make clear what criteria were used to determine the sample size. The main points here are to (a) do what you reasonably can to attain adequate power and (b) explain how the number of participants was determined.

2. Multiple NHST tests inflate null-hypothesis rejection rates. Tests of statistical significance (e.g., t-tests, analyses of variance) should not be used repeatedly on different subsets of the same data set (e.g., on varying numbers of participants in a study) without statistical correction, because the Type I error rate increases across multiple tests.

A. One concern is the practice of testing a small sample of participants and then analyzing the data and deciding what to do next depending on whether the predicted effect (a) is statistically significant (stop and publish!), (b) clearly is not being obtained (stop, tweak, and start a new experiment), or (c) looks like it might become significant if more participants are added to the sample (test more

participants, then reanalyze; repeat as needed). If this “optional stopping rule” has been followed without appropriate corrections, then report that fact and acknowledge that the Type I error rate is inflated by the multiple tests. Depending on the views of the Editor and reviewers, having used this stopping rule may not preclude publication, but unless appropriate corrections to the Type I error rate are made it will lessen confidence in the reported results. Note that Bayesian data analysis methods are less sensitive to problems related to optional stopping than NHST methods.

B. It is problematic to analyze data and then drop some participants or some observations, re-run the analyses, and then report only the last set of analyses. If participants or observations were eliminated, then explicitly indicate why, when, and how this was done and either (a) report or synopsize the results of analyses that include all of the observations or (b) explain why such analyses would not be appropriate.

C. Covariate analyses should either be planned in advance or be described as exploratory. It is inappropriate to analyze data without a covariate, then re-analyze those same data with a covariate and report only the latter analysis as confirmation of an idea. It may be appropriate to conduct multiple analyses in exploratory research, but it is important to report those analyses as exploratory and to acknowledge possible inflations of the Type I error rate.

D. If multiple dependent variables (DVs) are individually analyzed with NHST, the probability that at least one of them will be “significant” by chance alone grows with the number of DVs. Therefore it is important to inform readers of all of the DVs collected that are relevant to the study. For example, if accuracy, latency, and confidence were measured, but the paper focuses on the accuracy data, then report the existence of the other measures and (if possible) adjust the analyses as appropriate. Similarly, if several different measures were used to tap a construct, then it is important to report the existence of all of those indices, not just the ones that yielded significant effects (although it may be reasonable to present a rationale for why discounting or not reporting detailed results for some of the measures is justified). There is no need to report measures that were available to you (e.g., via a participant pool data base) but that are irrelevant to the study.

3. Rich descriptions of the data help reviewers, the Editor, and other readers understand your findings. Thus it is important to report appropriate measures of variability around means and around effects (e.g., confidence intervals around means and/or around standardized effect sizes).

4. Cherry picking experiments, conditions, DVs, or observations can be misleading. Give readers the information they need to gain an accurate impression of the reliability and size of the effect in question.

A. Conducting multiple experiments with the same basic procedure and then reporting only the subset of those studies that yielded significant results (and putting the other experiments in an unpublished “file drawer”) can give a misleading impression of the size and replicability of an effect. If

several experiments testing the same hypothesis with the same or very similar methods have been conducted and have varied in the pattern of significant and null effects obtained (as would be expected, if only due to chance), then you should report both the significant and the non-significant findings. Reporting the non-significant findings can actually strengthen evidence for the existence of an effect when meta-analytical techniques pool effect sizes across experiments. It is not generally necessary to report results from exploratory pilot experiments, such as when pilot experiments were used to estimate effect size, provided the final experiment has high power. In contrast, it is not appropriate to run multiple low-powered pilot experiments on a given topic and then report only the experiments that reject the null hypothesis.

B. Deciding whether or not to report data from experimental conditions post hoc, contingent on the outcome of NHST, inflates the Type I error rate. Therefore, please inform readers of all of the conditions tested in the study. If, for example, 2nd, 4th, and 6th graders were tested in a study of memory development then it is appropriate to report on all three of those groups, even if one of them yielded discrepant data. This holds even if there are reasons to believe that some data should be discounted (e.g., due to a confound, a ceiling or floor effect in one condition, etc.). Here again, anomalous results do not necessarily preclude publication (after all, even ideal procedures yield anomalous results sometimes by chance). Failing to report the existence of a condition that did not yield the expected data can be misleading.

C. Deciding to drop participants or observations post hoc contingent on the outcome of NHST inflates the Type I error rate. Best practice is to set inclusion/exclusion criteria in advance and stick to them, but if that is not done then whatever procedure was followed should be reported.

5. Be careful about using null results to infer “boundary conditions” for an effect. A single experiment that does not reject the null hypothesis provides only weak evidence for the absence of an effect. Too much faith in the outcome of a single experiment can lead to hypothesizing after the results are known (HARKing), which can lead to theoretical ideas being defined by noise in experimental results. Unless the experimental evidence for a boundary condition is strong, it may be more appropriate to consider a non-significant experimental finding as a Type II error. Such errors occur at a rate that reflects experimental power (e.g., if power is .80, then 20% of exact replications would be expected to fail to reject the null).

6. Authors should use statistical methods that best describe and convey the properties of their data. The Psychonomic Society does not require authors to use any particular data analysis method. The following sections highlight some important considerations.

A. Statistically significant findings are not a prerequisite for publication in Psychonomic Society journals. Indeed, too many significant findings relative to experimental power can indicate bias.

Sometimes strong evidence for null effects can be deeply informative for theorizing and for identifying boundary conditions of an effect.

B. In many scientific investigations the goal of an experiment is to measure the magnitude of an effect with some degree of precision. In such a situation a hypothesis test may be inappropriate as it only indicates whether data appear to differ from some specific theoretical value. Sometimes stronger scientific arguments can be made with confidence intervals (of parameter values or of standardized effect sizes). Moreover, some of the bias issues described above can be avoided by designing experiments to measure effects to a desired degree of precision (range of confidence interval).

C. The Psychonomic Society encourages the use of data analysis methods other than NHST when appropriate. For example, Bayesian data analysis methods avoid some of the problems described above. They can be used instead of traditional NHST methods for both hypothesis testing and estimation.

Last Word

Ultimately, journal Editors work with reviewers and authors to promote good scientific practice in publications in Psychonomic Society journals. A publication decision on any specific manuscript depends on much more than the above guidelines, and individual Editors and reviewers may stress some points more than others. Nonetheless, all else being equal submissions that comply with these guidelines will be better science and be more likely to be published than submissions that deviate from them.

Resources

There are many excellent sources for information on statistical issues. Listed below are some that the 2012 Publications Committee and Editors recommend.

Confidence Intervals:

Cumming, G. (2012). Understanding the new statistics: Effect sizes, confidence intervals, and meta-analysis. New York, NY US: Routledge/Taylor & Francis Group. (see www.latrobe.edu.au/psy/research/projects/esci).

Masson, M. J., & Loftus, G. R. (2003). Using confidence intervals for graphically based data interpretation. Canadian Journal of Experimental Psychology/Revue Canadienne de Psychologie Expérimentale, 57, 203-220. doi:10.1037/h0087426

Effect Size Estimates:

Ellis, P. D. (2010). The essential guide to effect sizes: Statistical power, meta-analysis and the interpretation of research results. Cambridge University Press. ISBN 978-0-521-14246-5.

Fritz, C. O., Morris, P. E., & Richler, J. J. (2011). Effect size estimates: Current use, calculations and interpretation. Journal of Experimental Psychology: General, 141, 2-18.

Grissom, R. J., & Kim, J. J. (2012). Effect sizes for research: Univariate and multivariate applications (2nd ed.). New York, NY US: Routledge/Taylor & Francis Group.

Meta-analysis:

Cumming, G. (2012). Understanding the new statistics: Effect sizes, confidence intervals, and meta-analysis. New York, NY US: Routledge/Taylor & Francis Group. (see www.latrobe.edu.au/psy/research/projects/esci).

Littell, J. H., Corcoran, J., & Pillai, V. (2008). Systematic reviews and meta-analysis. New York: Oxford University Press.

Bayesian Data Analysis:

Kruschke, J. K. (2011). Doing Bayesian data analysis: A tutorial with R and BUGS. San Diego, CA US: Elsevier Academic Press. (See www.indiana.edu/~kruschke/DoingBayesianDataAnalysis/)

Kruschke, J. K. (in press). Bayesian estimation supersedes the t test. Journal of Experimental Psychology: General. For a preprint see http://www.indiana.edu/~kruschke/BEST/BEST.pdf .

Power Analysis

Faul, F., Erdfelder, E., Lang, A., & Buchner, A. (2007). G*Power 3: A flexible statistical power analysis program for the social, behavioral, and biomedical sciences. Behavior Research Methods, 39(2), 175-191. (See http://www.psycho.uni-duesseldorf.de/abteilungen/aap/gpower3/)
