# Supplementary material for: The influence of journal submission guidelines on authors' reporting of statistics and use of open research practices
Source: PLoS One. 2017 Apr 17;12(4):e0175583. doi: 10.1371/journal.pone.0175583 (PMC5393581; doi:10.1371/journal.pone.0175583)
Supplement: S3 File — (PDF) [file pone.0175583.s003.pdf]

S3 File. PLOS ONE data availability

Retrieved from: http://journals.plos.org/plosone/s/data-availability (Accessed March 2017).

PLOS journals require authors to make all data underlying the findings described in their manuscript fully available without restriction, with rare exception.

When submitting a manuscript online, authors must provide a *Data Availability Statement* describing compliance with PLOS's policy. If the article is accepted for publication, the data availability statement will be published as part of the final article.

Refusal to share data and related metadata and methods in accordance with this policy will be grounds for rejection. PLOS journal editors encourage researchers to contact them if they encounter difficulties in obtaining data from articles published in PLOS journals. If restrictions on access to data come to light after publication, we reserve the right to post a correction, to contact the authors' institutions and funders, or in extreme cases to retract the publication.

Methods acceptable to PLOS journals with respect to data sharing are listed below, accompanied by guidance for authors as to what must be indicated in their data availability statement and how to follow best practices in reporting. If authors did not collect data themselves but used another source, this source must be credited as appropriate. Authors who have questions or difficulties with the policy, or readers who have difficulty accessing data, are encouraged to contact the relevant journal office or data@plos.org.

The data policy was implemented on March 3, 2014. Any paper submitted before that date will not have a data availability statement. However for all manuscripts submitted or published before this date, data must be available upon reasonable request.
