# Supplementary material for: The influence of journal submission guidelines on authors' reporting of statistics and use of open research practices
Source: PLoS One. 2017 Apr 17;12(4):e0175583. doi: 10.1371/journal.pone.0175583 (PMC5393581; doi:10.1371/journal.pone.0175583)
Supplement: S4 File — (PDF) [file pone.0175583.s004.pdf]

S4 File. Psychological Science Statistical Guidelines

Retrieved from: http://www.psychologicalscience.org/publications/psychological_science/ps-submissions#STAT (Accessed March 2017)

Statistics

Effective January 2014, Psychological Science recommends the use of the “new statistics”—effect sizes, confidence intervals, and meta-analysis—to avoid problems associated with null-hypothesis significance testing (NHST). Authors are encouraged to consult this Psychological Science tutorial by Geoff Cumming, which shows why estimation and meta-analysis are more informative than NHST and how they foster development of a cumulative, quantitative discipline. Cumming has also prepared a video workshop on the new statistics that can be found here.

In previous editions of the journal’s Submission Guidelines, authors were advised: “Effect sizes should accompany major results. When relevant, bar and line graphs should include distributional information, usually confidence intervals or standard errors of the mean.” Now, with the emphasis on estimation, authors should continue to include effect sizes for their major results and distributional information in their graphs (or tables, for that matter). However, authors are strongly advised to report 95% confidence intervals instead of standard deviations or standard errors, because confidence intervals convey much more useful information—another key point discussed at length in Cumming’s tutorial.

Reporting Statistical Results

To promote transparency and precision, authors should report test statistics with two decimal points (e.g., $t(34)=5.67$) and probability values with three decimal points. In addition, exact p-values should be reported for all results between .001 and .249; values below this range should be described as “$p<.001$” and those above as “$p>.250$.” Authors should be particularly attentive to APA style when typing statistical details (e.g., Ns for chi-square tests, formatting of dfs), and if special mathematical expressions are required, they should not be graphic objects but rather inserted with Word’s Equation Editor or similar.
