# Supplementary material for: The influence of journal submission guidelines on authors' reporting of statistics and use of open research practices
Source: PLoS One. 2017 Apr 17;12(4):e0175583. doi: 10.1371/journal.pone.0175583 (PMC5393581; doi:10.1371/journal.pone.0175583)
Supplement: S5 File — (PDF) [file pone.0175583.s005.pdf]

S5 File. Journal of Experimental Psychology: General Guidelines

Retrieved from: http://www.apa.org/pubs/journals/xge/?tab=4 (Accessed 2017)

In addition, APA Ethical Principles specify that "after research results are published, psychologists do not withhold the data on which their conclusions are based from other competent professionals who seek to verify the substantive claims through reanalysis and who intend to use such data only for that purpose, provided that the confidentiality of the participants can be protected and unless legal rights concerning proprietary data preclude their release" (Standard 8.14).

APA expects authors to adhere to these standards. Specifically, APA expects authors to have their data available throughout the editorial review process and for at least 5 years after the date of publication.
