# Supplementary material for: The influence of journal submission guidelines on authors' reporting of statistics and use of open research practices
Source: PLoS One. 2017 Apr 17;12(4):e0175583. doi: 10.1371/journal.pone.0175583 (PMC5393581; doi:10.1371/journal.pone.0175583)
Supplement: S7 File — (PDF) [file pone.0175583.s007.pdf]

S7 File. Clinical psychological science results

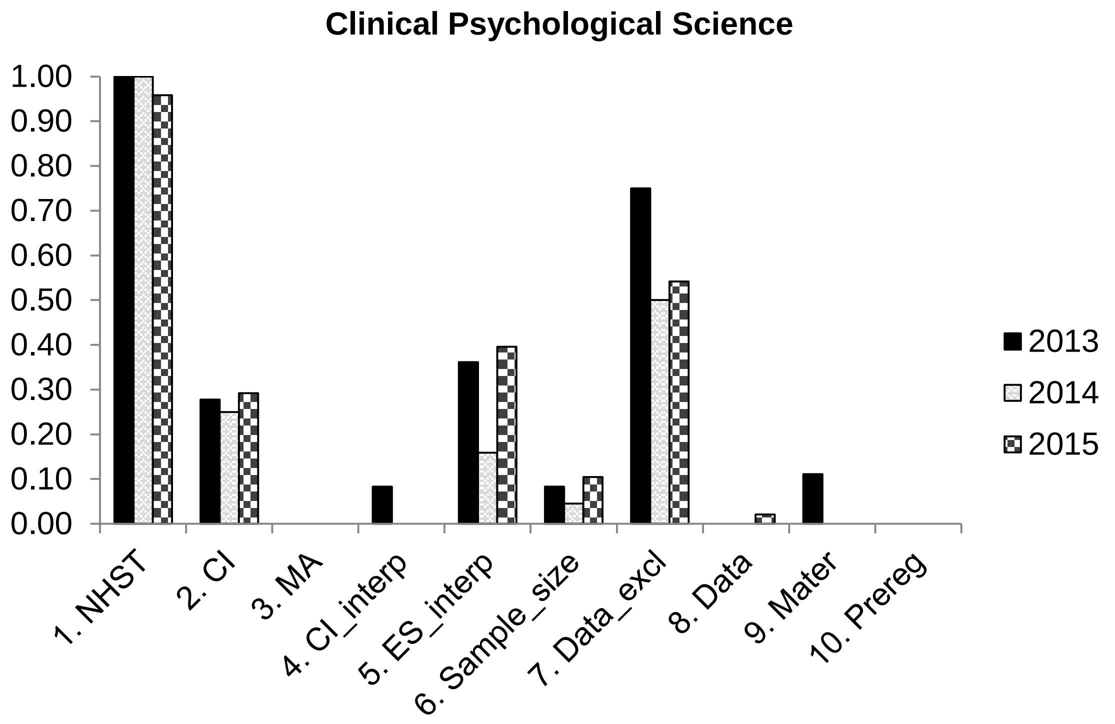

Figure 1S. Proportions of papers with at least one occurrence of various practices, in Clinical *Psychological Science*, for each of three years, see text for definitions of labels on the horizontal axis. Number of papers included: 2013 (n = 36), 2014 (n = 44), 2015 (n = 48).

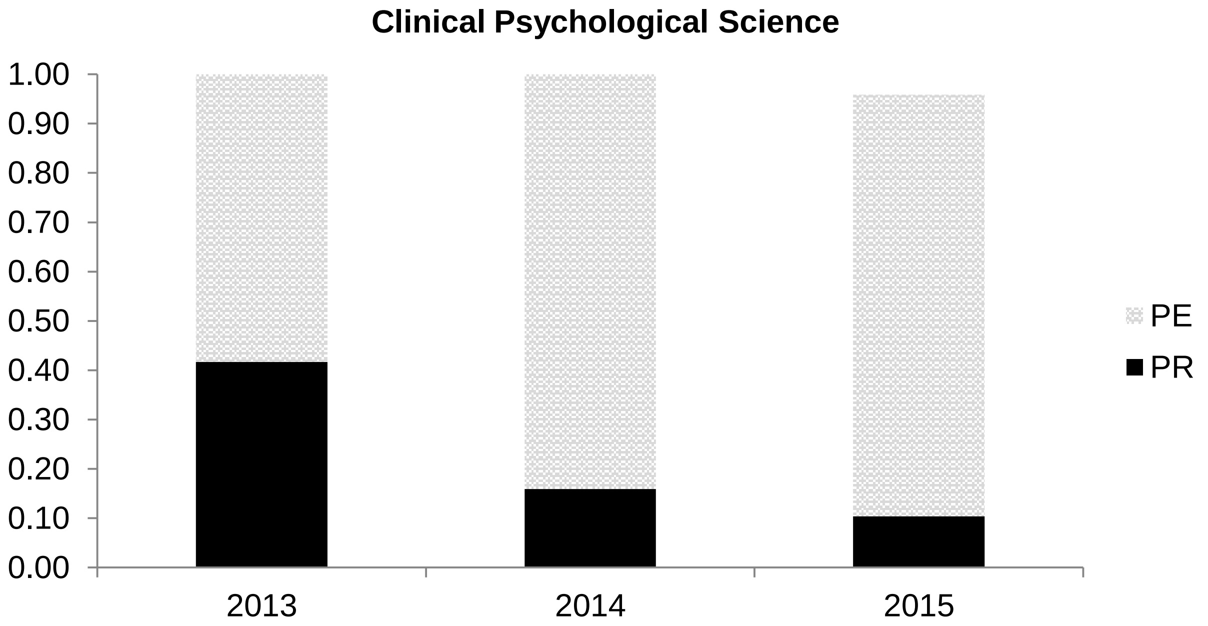

Figure 2S. Proportions of papers with PR (*p* relative) or PE (*p* exact), in Clinical *Psychological Science* for each of three years.
