# Supplementary material for: The influence of journal submission guidelines on authors' reporting of statistics and use of open research practices
Source: PLoS One. 2017 Apr 17;12(4):e0175583. doi: 10.1371/journal.pone.0175583 (PMC5393581; doi:10.1371/journal.pone.0175583)
Supplement: S1 Text — (PDF) [file pone.0175583.s008.pdf]

S1 Text. Changes with respect to the preregistered study.

After having collected all the data in accordance with the criteria of the preregistered study, we decided to make major changes in the dataset. The scoring criteria have been changed for clarity and practical purposes.

We initially considered separately the CI and the effect size CI. However, this distinction generated some confusion and we decided to combine CI and effect size CI into a single category.

We initially also considered where CI or effect sizes were reported (table, text and figures). This generated some confusion, and we decided to do not include this distinction.

Standardized effect sizes were originally considered. For clarity and to avoid possible confusion between the ES and the ES_interpr practices we decided to do not report these data.

We considered all of the papers that appeared in *Clinical Psychological Science* (*CPS*) between 2013 and 2015. However, the comparison with *CPS* was problematic. In fact, *CPS* is a very young journal and publishes articles having to do with clinical psychology, unlike *Psychological Science* and the *Journal of Experimental Psychology: General*. Another reason is that CPS publishes typically studies with clinical populations that are often subject to more stringent privacy issues that preclude the release of data. Therefore, we decided to report the *CPS* data only in the Supplemental Material and to avoid to comparing this journal with *Psychological Science* or the *Journal of Experimental Psychology: General*.

Use of the NA category: Many papers were originally coded as NA. In fact, the use of this category was somewhat problematic. We therefore have decided to do not use the NA category when possible, and NA was only used for meta-analyses.
