# Supplementary material for: The influence of journal submission guidelines on authors' reporting of statistics and use of open research practices
Source: PLoS One. 2017 Apr 17;12(4):e0175583. doi: 10.1371/journal.pone.0175583 (PMC5393581; doi:10.1371/journal.pone.0175583)
Supplement: S2 Text — (PDF) [file pone.0175583.s009.pdf]

S2 Text. Examples of journals offering the option to submit pre-study peer review

Emotion and Cognition: no submission (Agneta Fischer, Dec 2015, personal communication); Cortex: 19 received, 2 published in 2015 (≈1%), (Christopher Chambers, Dec 2015, personal communication); Attention, Perception, & Psychophysics: 5 received, 1 published (≈ .5%), (Michael Dodd, Dec 2015, personal communication).
